# Influence of design implant and apical depth in post-extraction sockets: an in vitro simulated study

**DOI:** 10.1186/s12903-023-02999-9

**Published:** 2023-05-25

**Authors:** Marcelo Sales Cavalcante, Marcelo Ferraro-Bezerra, Paulo Goberlanio de Barros Silva, Gabriel Silva Andrade, Phillipe Nogueira Barbosa Alencar, Josfran da Silva Ferreira Filho, Lucas Alexandre Maia, Raul Anderson Domingues Alves da Silva, Danna Mota Moreira, Rafael Linard Avelar

**Affiliations:** 1 School of Dentistry, Center University Christus, 133, Adolfo Gurgel Street, Fortaleza, CE Brazil; 2 Department of Clinical Dentistry, Oral and Maxillofacial Surgery Service, Federal University of Ceará, Walter Cantídio University Hospital, Fortaleza, Brazil; 3 Oral and Maxillofacial Surgery Resident at Institute Jose Frota Hospital, Fortaleza, Brazil

**Keywords:** Implant, Design, Initial stability, Tapered implants

## Abstract

**Background:**

Implant design and apical stability are principal parameters involved in achieving successful primary stability. Using polyurethane models to simulate post-extraction sockets, we investigated the effects of using differing blade designs on the primary stability of tapered implants and the impact of apical depth.

**Method:**

Six polyurethane blocks were used to simulate post-extraction pockets. One of the implants presented self-tapping blades (Group A), while the other (Group B) did not. Seventy-two implants were placed at 3 different depths (5 mm, 7 mm, and 9 mm), and a torque wrench was used to measure the stability of the implants.

**Results:**

When evaluating the implants (placed at 5 mm, 7 mm, and 9 mm apical to the socket), we observed that the torque of the Group B implants was higher than that of Group A implants (*P* < 0.01). At the 9-mm depth, there was no difference between the groups (Drive GM 34.92 Ncm and Helix GM 32.33 Ncm) (*P* > 0.001), and considering the same implant groups, those placed at 7-mm and 9-mm depths presented higher torques (*p* < 0.01) than those placed at 5-mm (*p* > 0.01).

**Conclusion:**

Considering both groups, we concluded that an insertion depth of greater than 7 mm is needed for initial stability, and in situations involving reduced supportive bone tissue or low bone density, a non-self-tapping thread design improves implant stability.

**Supplementary Information:**

The online version contains supplementary material available at 10.1186/s12903-023-02999-9.

## Background

The introduction of osseointegrated implants has marked a turning point in dental practice. Immediate loading of dental implants has become more popular, which is due to several factors that include reduced treatment time, and aesthetic and psychological benefits for the patient. A fundamental prerequisite for implant success is primary stability at insertion [1, 2]. This can be indirectly measured by recording insertion torque data or by measuring the implant stability quotient (ISQ) using resonance frequency analysis (RFA) [2]. Studies have shown that primary stability is influenced by factors such as implant length and diameter, design, insertion technique, and compatibility between the implant and the surrounding bone [3–5]. Procedures for placement of implants in post-extraction sockets require excellent apical stabilization [6], while both bone quality [2] and quantity remain key stabilization factors. Low bone density and/or reduced supporting bone tissue remain the greatest risk factors for implant loss [3]. With regard to design, tapered implants are reported to be more stable than cylindrical implants [6–8]. Implant stability is also associated with thread design, and thus thread depth, thickness, angle, pitch, and helix angle all affect the biomechanical loading distribution of the implant [2, 9]. The success of immediate loading protocols depends strictly on the clinician’s ability to ensure the stability of the primary implant and then to monitor changes in stability and healing time. Our study, based on the hypothesis that implants of different shapes and placed at greater depths may provide greater primary stability, (allowing implants to receive an occlusal load earlier), evaluated the mechanical stability of tapered implants, when placed in simulated anterior maxilla region post-extraction bone cavities.

## Methods

A controlled in vitro laboratory mechanical test regime involving 72 titanium tapered implants (dimension: 3.5 × 13 mm) was performed (Neodent, Brazil). The implants were not experimental; i.e., they were available on the market at the time: “Drive” and “Helix”. The implants presented two macrogeometries with different thread designs (Groups A and B). Group A implants presented tapered bodies, with cylindrical coronal portions, and tapered apical portions. The apices were active and included smooth, rounded tips, and helical chambers. The threads were compressive in the coronal portions, and triangular and self-cutting in the apical regions. Group B implants presented double square main threads, with counterclockwise cutting chambers distributed along the implant bodies, and rounded apices with cutting edges.

**Fig. 1:**
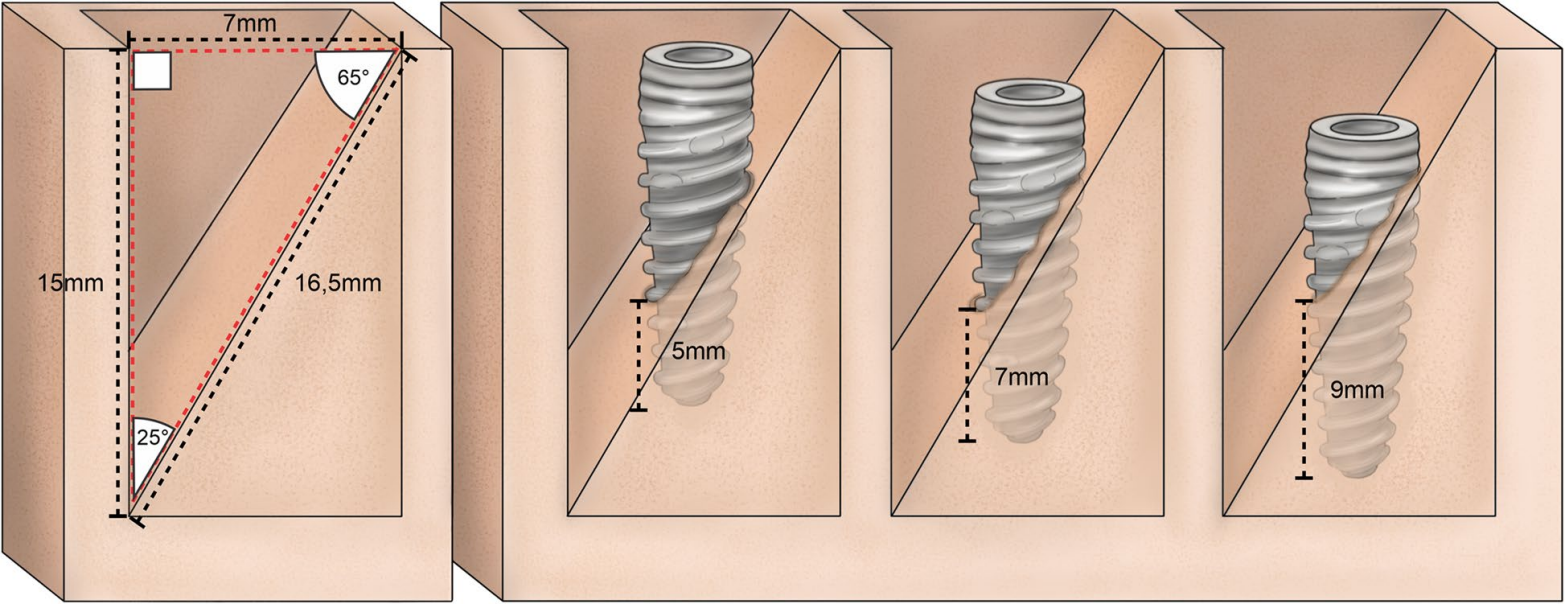
Diagram of an implant placed in a “step” osteotomy (Groups)

**Fig. 2:**
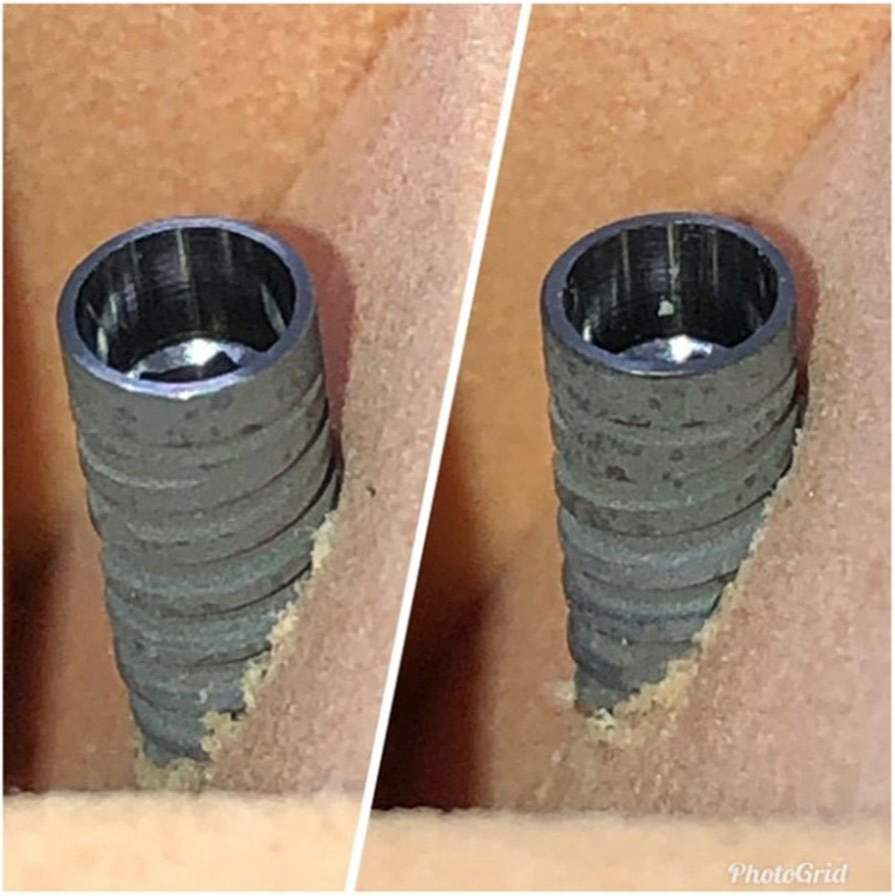
Implants placed without lateral contacts (“step” osteotomies) in dense artificial bone, representing implant placement in a fresh extraction socket with only apical stability. GM™ Drive™ implants placed at 5 mm and 7 mm, respectively

Six polyurethane blocks (Nacional Ossos, Jaú, São Paulo, Brazil) were used, presenting the following specifications: 60 mm wide × 140 mm long × 33 mm thick. The block density was PCF 30, corresponding to 0.48 g/cm^3^, and simulated bone conditions in the anterior maxillary region. Twelve pyramidal simulations of the upper central incisor sockets (Fig. 1) were performed with the insertion of 12 implants (1 in each simulation). The simulations are demonstrated in Fig. 2.

To simulate post-extraction sockets, twelve defects were created in the 6 polyurethane blocks produced during the manufacture of the material. The defects in these sockets are presented in Fig. 2. All simulations were performed by the same surgeon, with extensive experience in implant dentistry. Two types of implants were evaluated: 36 Helix Gran Morse implants (Group A), and 36 Drive Gran Morse implants (Group B); both produced by Neodent, Curitiba, Brazil. The implants presented similar patterns, 3.5-mm diameters, and 13-mm heights.

Drive implants are most commonly used for type III and IV bones and allow for a greater bone expansion. Helix implants are generally used for denser bones (type I and II), and present greater drilling capacity. The milling sequence used for the implants was: drill bit, 2-mm helical drill, pilot drill for cervical enlargement, and a 3.3-mm tapered drill at 5-mm, 7-mm, 9-mm depths (Fig. 2), observing the depths of 3 mm, 5 mm, 7 mm. The bits were marked to define drilling depth.

### Implant insertion torque measurement

**Fig. 3:**
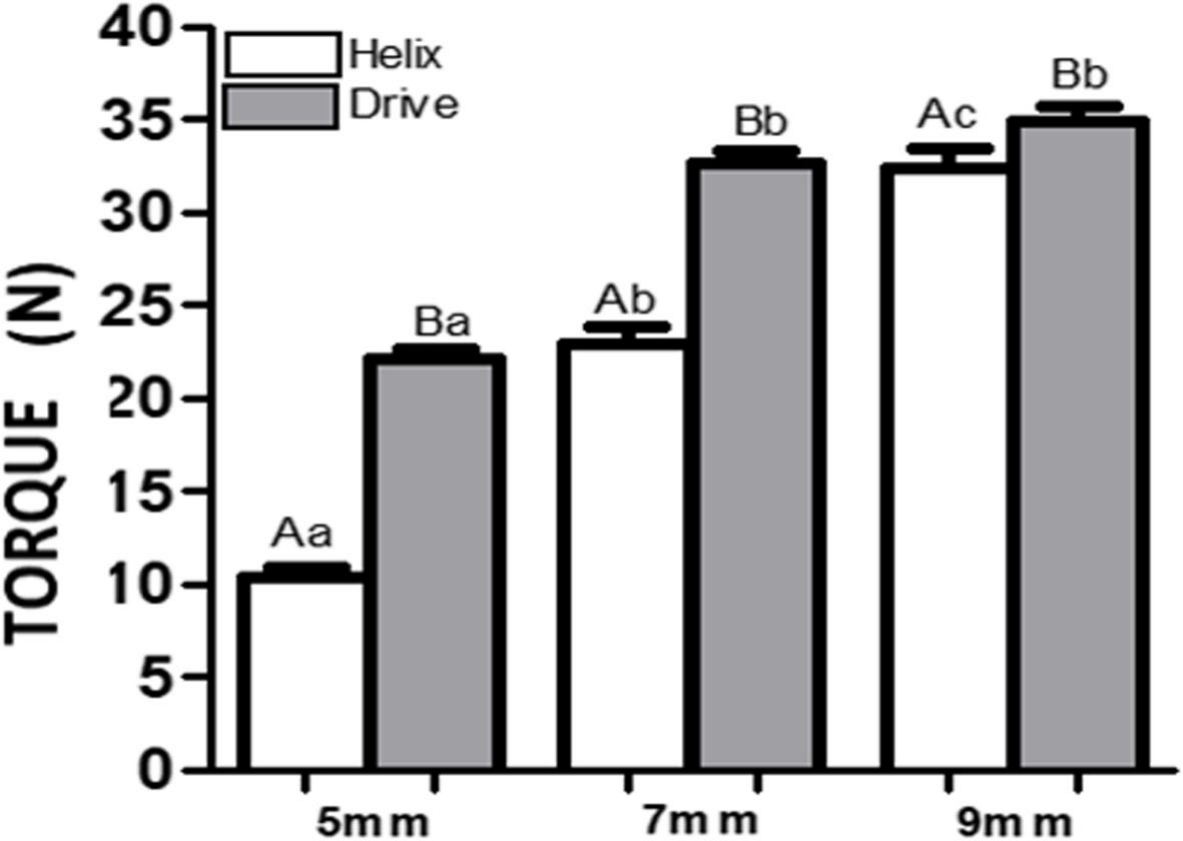
Insertion torque of implants at different depths

**Fig. 4:**
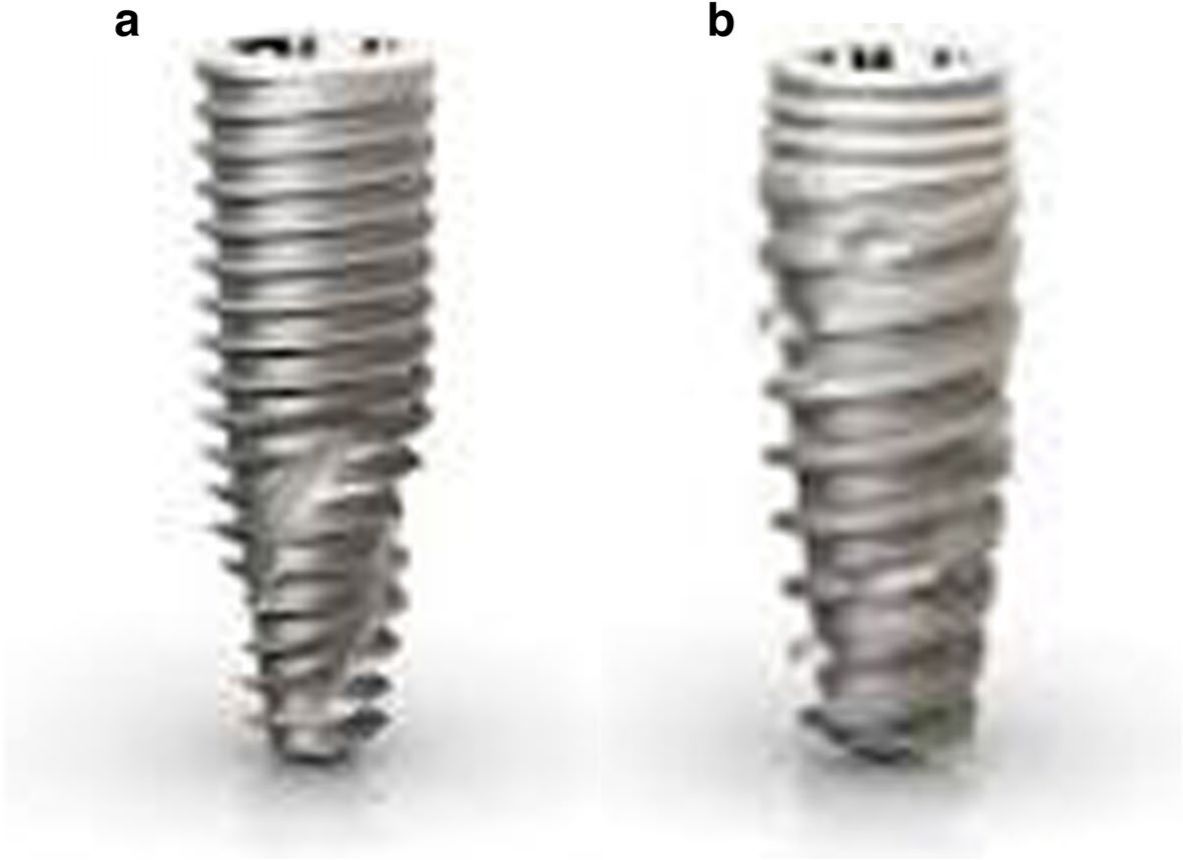
**a** and **b** Dental implants used as Group A (Helix) – **a** and Group B (Drive) – **b**

A TQ 680 digital torque wrench, manufactured by Instrutherm, Instrumentos de Medição Ltda, (São Paulo, SP, Brazil), was used for measuring the insertion torque. The torque wrench was attached to an adjustable guiding base using a spindle and a hand wrench, which allowed it to be stably positioned at 90º angle. After placing the implant at a predetermined height, a hand wrench (Neodent Grand Morse Implant Kit), was placed in the torque wrench spindle, and was then adjusted for rigid attachment. The implant head was then connected to the insertion wrench, and the procedure for measuring the implant torque began with the torque wrench being turned clockwise, with same surgeon noting the values indicated on the equipment. The electronic equipment, consisting of a digital torque wrench (Tohnichi Series QSP Model QSP25N3) also connected to a computer for reading the peak value of insertion torque every millisecond, was customized for this study. The resulting values were then compared (Fig. 3). The dental implants used as Group A (Helix) and Group B (Drive) (Fig. 4a and b).

### Statistical analysis

The data, expressed as mean and standard error, were subjected to the Kolmogorov–Smirnov normality test, and compared using the Mann–Whitney and Kruskal–Wallis/Dunn tests (Non-parametric data) (*p* < 0.05), GraphpAdPrism5.0).

## Results

As shown in Table 1, the insertion torque of the implants was first obtained at different depths, followed by the calculation of the mean standard deviations for the Group (A and B) data at 5-mm, 7-mm, and 9-mm depths. It can be observed in the column for implants placed at 5 mm apical to the socket that the torque of Group B implants was significantly higher than that of Group A implants (*P* < 0.01). The same was observed in the column of the implants placed 7 mm apical to the socket, since the internal value torque of Group B implants (32.67 Ncm) was significantly higher than that of Group A (22.83 Ncm). When we compared the groups at the 9 mm depth, we observed no significant difference between them (Drive GM 34.92 Ncm and Helix GM 32.33 Ncm) (*P* > 0.001). When analyzing Group A implants at 5-mm, 7-mm, 9-mm depths, the results reveal that the insertion torque was significantly lower at the 5-mm (10.42 Ncm) depth than at the 7-mm (22.83 Ncm) and 9-mm (32.33 Ncm) depths. Although in the Group B implants, the insertion torque at the 5-mm depth was lower than at the 7-mm depth (22.08 Ncm vs 32.67 Ncm) (*P* < 0.01). No statistically significant differences were observed between the 7-mm and 9-mm depths (32.67 Ncm vs 34.92 Ncm) (*P* > 0.01).

**Table 1:** Insertion torque of different implants at different depths

	**Depth**			***P*-value**^**†**^
	**5 mm**	**7 mm**	**9 mm**	
**Model**				
Group A	10.42 ± 0.51^Aa^	22.83 ± 0.98^Ab^	32.33 ± 1.12^Ac^	** < *.001***
Group B	22.08 ± 0.58^Ba^	32.67 ± 0.64^Bb^	34.92 ± 0.72^Bb^	** < *.001***
*P*-Value*	** < *.001***	** < *.001***	***0.009***	

Capital letters = significant difference between groups; Lower case letters = significant difference between depths

Source: Author

^*^Test Mann–Whitney

^†^Kruskal–Wallis/Dunn Test (mean ± SEM)

## Discussion

The mechanical stability of the implant at the time of insertion, defined as primary stability, is one of the most important factors for implant success. Micro-movement at the bone-implant interface that exceeds a threshold of 50 to 150 µm can lead to the formation of fibrous instead of bone tissue. This impairs osseointegration. It was found that with immediate loading of the implants, insertion torque scores below 20 Ncm were indicative of higher failure rates [10]. According to Romanos [6], the apical portion may play an important role in implant stability, and based on current data, the apical third of the implant length contributes to between 30 and 43% of the stability of the entire implant [6]. This was also observed in the present study, which showed that insertion torque is greater with greater apical depth, and implants positioned at more than 7-mm deep may permit clinically safe immediate loading. Chang et al. [11] investigated the effect of self-tapping blades on the initial stability of tapered implants in polyurethane bone blocks. Their findings indicated that tapered implants without self-tapping blades have the same primary stability as implants with self-tapping blades when 1/3 to 2/3 covered by bone, which differs from the results of our study. In our study, 30 to 50% of the implant was submerged, and the Drive GM Implant (Group B), with square and double primary threads, presented a higher insertion torque for all measurements at the same depths, than the Helix GM implant (Group A), which possessed compressive trapezoidal threads in the coronal portion, and self-drilling-self-tapping triangular threads in the apical region. Threads maximize initial contact, improve initial stability, increase implant surface area, and favor stress dissipation throughout the implant. The standard V-shaped thread promotes 10 times more shear loading on the bone than a square thread with similar diameters [9, 12]. Hybrid implant designs present both conical and cylindrical segments, and at 5 mm, the possibility of locking (in both portions) favors the stability of the implant. Falco et al. [2] observed that implants with large and self-cutting blades display greater primary stability than implants with small blades, especially in cases of low quantity or poor quality bone. If only a few millimeters of apical bone are available to stabilize the implant (as in post-extraction implants), the implant macrogeometry becomes critical for achieving sufficient primary stability. Three-dimensional optical tests using different implant thread geometries has revealed increased apical direction stress in implants with lower coronal region stress. Elastic studies reveal that the geometry of the apical thread is a key factor in bone remodeling, and support the hypothesis that new bone formation results from loading forces acting in this region of the implant [8, 13].

In cases of low bone density, incorrect implant geometry may result in insufficient stability for immediate loading [2]. This was observed in this study; implants with thinner blades (Group A) presented poorer results than Group B in all measures evaluated. Tapered implants were initially designed for immediate loading after dental extraction. Tapered implants provide cortical bone compression in regions with poor quality bone tissue, or in post-extraction sockets [1, 14]. Full-body cylindrical implants increase the risk of lip perforation because of buccal concavities; the decrease in the diameter of tapered implants towards the apical region takes lip concavity into consideration [1, 15]. Some studies have shown higher insertion torque values in tapered implants than in cylindrical implants, suggesting greater stability [14]. To reduce the shear force component, by moving axial loading from the prosthesis to the apex of the implant; a square-thread tapered implant is suggested. This would transfer more of the axial load throughout the implant body, and compress the bone [12]. The implants with square threads [9], such as Drive GM, presented higher success rates in simulations with post-extraction sockets. Obtaining primary stability during implant insertion is essential for achieving osteointegration throughout the entire healing phase. However, the performance of immediate loading procedures in post-extraction sockets depends on variables that are often difficult to assess, such as the general state of health of the patient, bone quality, the implant edge and material used, and the surgical ability of the surgeon [1, 5, 7].

There are various tests which evaluate implant stability, including percussion, digital pressure, radiographs, RF analysis (RFA) using the Periotest (Medizintechnik Gulden, Modautal, Germany), and insertion torque. According to Andreaza da Cunha et al. [11, 16], RFA and insertion torque methods are more efficient and present fewer contraindications [11]. Radio Frequency Analysis (RFA), which is measured using the Osstell device and insertion torque are the most often used test procedures [10, 11, 17]. An aggressive implant insertion with greater thread depth, which provides close contact between the implant surface and the bone, also generates insertion torque. If excessive force is used during implant insertion, the compression may exceed physiological limits and trigger bone resorption, leading to necrosis and implant failure [18]. The disadvantage of using insertion torque is that it is a single parameter which can only be measured once at the time of implant placement, while RFA can be performed during all phases of implant treatment [17, 19]. The Osstell device can be used at the time of implant placement, during the healing period, and when the dental prosthesis is in use [17]. However, it has the disadvantages of not providing an absolute value, and it does not allow comparison between the stabilities of different implants.

Insertion torque was used in this study because it is easy to manage, and it allows predicting results and comparing values at the time of implant placement, as well as comparing implants in the same bone conditions. The bone cavity model developed in this study demonstrated clinically realistic levels of insertion torque and implant stability, while simulating the low bone quality typically found at an extraction site. Moreover, this model enabled simulating the presence of cortical bone on the inner side of the socket, which is an important clinical challenge faced when studying bone defects after extraction. As a limitation, this study did not address the relationship between density and depth of the placed implant, but rather the variation of implant depth when placed in post-extraction socket areas, (which is a very common condition in patients who present the need for both extraction and immediate placement of implants) and did not evaluate: marginal bone loss around neck implants, dental higyene procedures or microleakage and connection with dental prosthetics [20–23]. In this study, digital workflow can benefit the process of rehabilitation [21]. This is common in the anterior region of the maxilla, which has a density similar to the polyurethane block used in this study. When assessing bone depth, especially in post-extraction sockets, the apical portion of the implant contributes to implant stability. We conclude that for immediate loading, a minimum of 7 mm of apical anchorage is required, and that implants with square threads provide additional stability when placing immediate loading implants.

## Supplementary Information

**Additional file 1.**

## Data Availability

The datasets used and/or analyzed during the current study available. Contact Email: Rafael.avelar@unichristus.edu.br.
