# Supplementary figures and images for: Influence of design implant and apical depth in post-extraction sockets: an in vitro simulated study
Source: BMC Oral Health. 2023 May 25;23:322. doi: 10.1186/s12903-023-02999-9 (PMC10214678; doi:10.1186/s12903-023-02999-9)

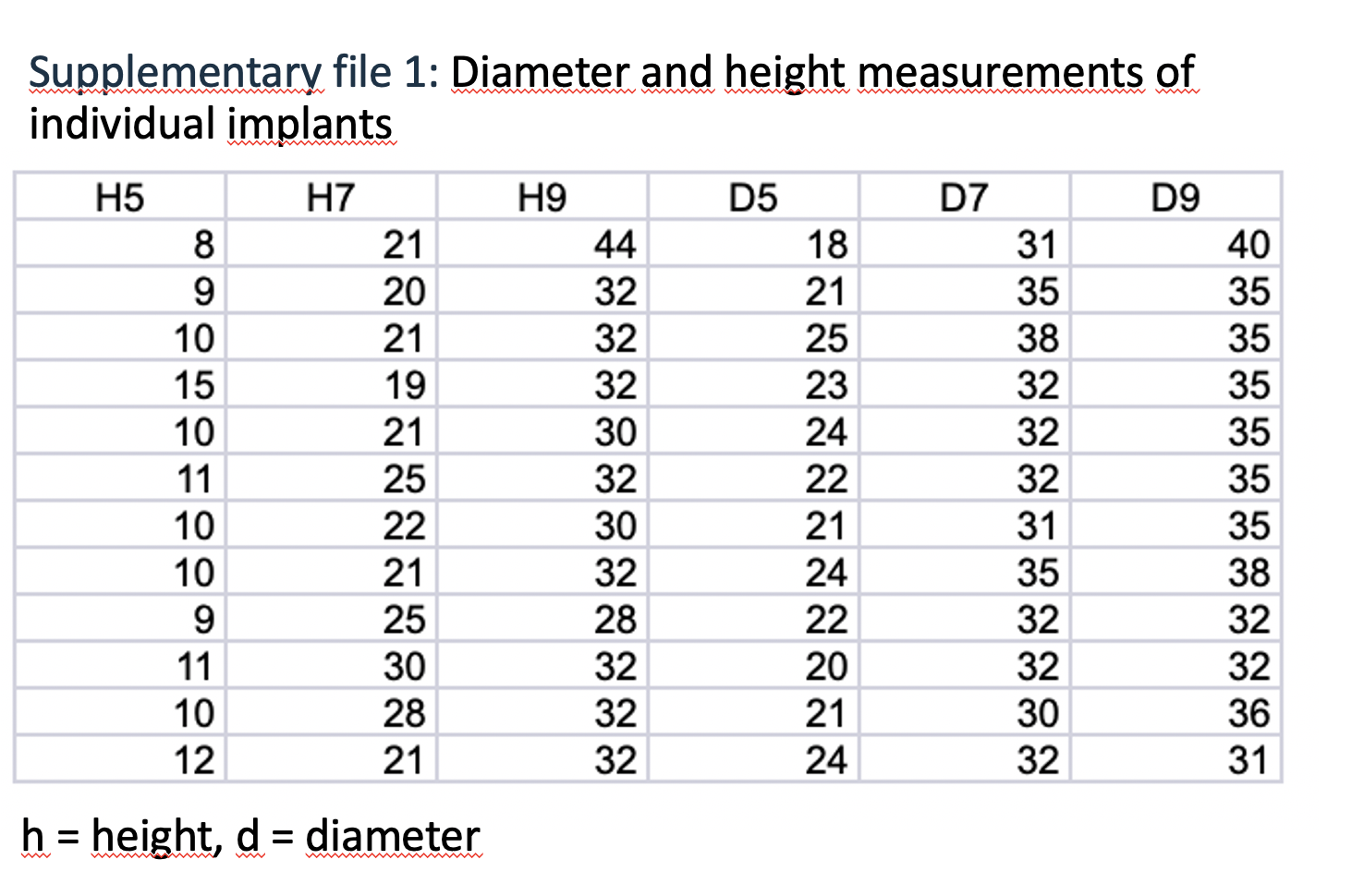

Supplement: Supplementary file 1 — Additional file 1. [file 12903_2023_2999_MOESM1_ESM.png]
